# Physiological and transcriptome analysis of changes in endogenous hormone and sugar content during the formation of tender asparagus stems

**DOI:** 10.1186/s12870-024-05277-0

**Published:** 2024-06-19

**Authors:** Maolin He, Peiran Chen, Mengyao Li, Fengyun Lei, Wei Lu, Chengyao Jiang, Junting Liu, Yanwen Li, Jiachang Xiao, Yangxia Zheng

**Affiliations:** 1 College of Horticulture, Sichuan Agricultural University, Chengdu, 611130 China; 2 Agricultural Equipment Research Institute, Chengdu Academy of Agricultural and Forest Sciences, Chengdu, 611130 China

**Keywords:** Asparagus, Stem elongation, Endogenous hormones, Sugar

## Abstract

Asparagus is a nutritionally dense stem vegetable whose growth and development are correlated with its quality and yield. To investigate the dynamic changes and underlying mechanisms during the elongation and growth process of asparagus stems, we documented the growth pattern of asparagus and selected stem segments from four consecutive elongation stages using physiological and transcriptome analyses. Notably, the growth rate of asparagus accelerated at a length of 25 cm. A significant decrease in the concentration of sucrose, fructose, glucose, and additional sugars was observed in the elongation region of tender stems. Conversely, the levels of auxin and gibberellins(GAs) were elevated along with increased activity of enzymes involved in sucrose degradation. A significant positive correlation existed between auxin, GAs, and enzymes involved in sucrose degradation. The ABA content gradually increased with stem elongation. The tissue section showed that cell elongation is an inherent manifestation of stem elongation. The differential genes screened by transcriptome analysis were enriched in pathways such as starch and sucrose metabolism, phytohormone synthesis metabolism, and signal transduction. The expression levels of genes such as *ARF*, *GA20ox*, *NCED*, *PIF4*, and otherswere upregulated during stem elongation, while *DAO*, *GA2ox*, and other genes were downregulated. The gene expression level was consistent with changes in hormone content and influenced the cell length elongation. Additionally, the expression results of RT-qPCR were consistent with RNA-seq. The observed variations in gene expression levels, endogenous hormones and sugar changes during the elongation and growth of asparagus tender stems offer valuable insights for future investigations into the molecular mechanisms of asparagus stem growth and development and provide a theoretical foundation for cultivation and production practices.

**Supplementary Information:**

The online version contains supplementary material available at 10.1186/s12870-024-05277-0.

## Introduction

Asparagus (*Asparagus officinalis* L.) is a perennial herbaceous plant of the Liliaceae family and *Asparagus* genus, originating from the Mediterranean coast and Asia Minor. Its young stems are the edible parts. Asparagus is considered a nutritious food due to its low-caloric content, high fiber content, and presence of various phytochemicals such as fructose, flavonoids, vitamins, saponins, or cinnamic acid [1]. These constituents are reported to have various biological activities like stress relief [2], anti-cancer [3], and so on.

Elongation and growth of plant stem depend on cell growth and cell cycle regulation. At the cellular level, it mainly manifests as an increase in length and number of cells [4]. Endogenous hormones are important in plant stem growth and development [5]. The effects of auxin and gibberellins (GAs) on the elongation of plant stems have been elucidated in plants such as Arabidopsis [6], bamboo [7], and peanut [8]. Li et al. [9]. showed a negative correlation between ABA content and rapidly high growth of bamboo shoots. Hong et al. [10]. found that ABA treatment significantly shortened the stem length of rice. Sugar is an important carbohydrate found in plants, serving multiple functions, including providing energy to the organism, regulating cellular osmotic pressure, and participating in signaling [11]. Sucrose (Suc), the main photosynthetic product of higher plants and the main form of sugar transport and storage, plays a crucial role in various stages of plant growth [12]. Sucrose synthase (SS) and invertase (INV) are mainly responsible for the sucrose degradation which operate subsequent to sucrose being unloaded from the phloem [13]. Chi et al. [14]. conducted an association analysis of the sorghum vacuolar invertase gene (*SbVIN1*), indicating a significant correlation between *SbVIN1* and stem length. A complex cross-reaction exists between hormones and sugar signaling [15]. Preliminary studies by Jia et al. [16]. and Moore et al. [17]. on Arabidopsis *hxk/gin2* showed that mutants exhibited resistance to exogenous IAA. Further studies demonstrated that the biological synthesis of IAA was induced by soluble sugars. According to the findings of Li et al. [18]. , Arabidopsis mutant ANAC060 has negative feedback on ABA signaling, leading to sugar insensitivity.

Based on asparagus transcription, Li et al. [19] identified and analyzed the NAC gene family, and Zhang et al. [20] elucidated its dynamic response mechanism to salinity stress, and so on. There is a certain understanding the physiological, biochemical, and molecular mechanisms underlying the elongation and growth of plant stems. However, there is a scarcity of research literature pertaining to the development of asparagus stems, a type of stem vegetable. This study employed the primary Sichuan asparagus variety ‘Fengdao 2’ as the experimental material. The elongation growth activity area was determined by observing the growth curve and tissue slices. Additionally, physiological indicators were measured and combined with transcriptome analysis to investigate further the internal mechanisms involving hormones and sugars in the elongation and growth of asparagus.

## Materials and methods

### Plant materials

This study used the three-year-old asparagus ‘Fengdao 2’, a high-quality produce with a large cultivation area. It is cultivated in the asparagus base (106.56° E, 31.65° N) of Enyang District, Bazhong City, Sichuan Province, with an altitude of approximately 580 m. Asparagus with a row spacing of 120 cm and a spacing of 30 cm is cultivated in rainproof greenhouses (6 × 50 m). From mid-March to mid-April 2022, 40 healthy and tender stems with a stem diameter of 13–15 mm (2 cm above the surface) were selected as the experimental material for cultivating asparagus mother stems. The height of the asparagus stems was measured using a steel ruler every morning, and this practice was stopped when the average value reached 150 cm. Select five asparagus plants with a height of 25 cm as four markers, and record the position of the markers within three days.

### Experimental treatment

Samples collection was started in early June 2022. The tender stems of ‘Fengdao 2’ with a stem diameter of 13–15 mm and plant heights of 10, 25, 40, and 60 cm were selected and designated as H-10, H-25, H-40, and H-60, respectively. For different growth and development stages of asparagus, 3–6 cm stem segments from the surface were collected from H-10, while 17–20 cm of stem segments from the surface were collected from H-25, H-40, and H-60 as the test materials. Each stage of asparagus contains 15 biological replicates. Tissue slice materials were placed in FAA fixed solution (50%) for storage and backup. Physiological indicator and transcriptome sequencing materials were stored in an ultra-low temperature refrigerator at − 80 ℃ for future use. Tissue sectioning, physiological indicators, transcriptome sequencing and RT-qPCR were repeated three times for each treatment.

### Physiological indicator measurement method

The determination of soluble sugar content was conducted using the anthrone colorimetric method. The determination of sucrose, glucose, and fructose content was improved based on the method proposed by Wu et al. [21]. The activities of sucrose synthase (decomposition direction; SS-I) and sucrose acid invertase (AI) were measured using the sucrose synthase (decomposition direction; SS-I) kit and soluble acid invertase (S-AI)/vacuolar invertase kit provided by Suzhou Grace Biotechnology Co., Ltd. The determination of auxin(IAA), GAs(GA_1_ + GA_3_ + GA_4_ + GA_7_), and ABA content was based on the HPLC-MS/MS method [22] and improved.

### Observation of asparagus tissue slices

The cell length of the central longitudinal section of the middle stem segment of asparagus was observed at different growth and development stages using the paraffin sectioning-microscopic observation method. Select 10 cells for statistical analysis in each period. The prepared sections were stained with Safranine-Solid Green and observed under a fully automatic Olympus DP70 microscope. Images were collected and edited using the CaseViewer microscopic image measurement and analysis software.

### Transcriptome sequencing and differentially expressed genes screening

Total RNA was extracted from asparagus tender stems using the Tiangen Polysaccharide Polyphenol Reagent Kit (QIAGEN, Germany) according to the manufacturer’s instructions. The RNA integrity was checked by calculating the RNA integrity number (RIN) using the Agilent Bioanalyzer 2100 (Agilent Technologies, Santa Clara, CA, USA). RNA samples were then sent to Novogene Biotech (Beijing, China) where strand-specific RNA-Seq library construction and sequencing were performed according to Illumina standard protocols. FPKM (one thousand base transcript fragments per million drawn) was employed as an indicator to measure transcript or gene expression levels. Differential expression was analyzed between sample groups using DESeq2 (version 1.20.0). Transcripts with a |log2 (FoldChange)| ≥ 1 and *p-value* ≤ 0.05 were identified as differentially expressed genes (DEGs). To obtain comprehensive gene function information on the biological functions and metabolic pathways represented by the DEGs, gene ontology (GO) and Kyoto encyclopedia of genes and genomes (KEGG) databases were used. GO was used to determine the distribution of gene functions at the macroscopic level. This database has three ontologies, namely, molecular function (MF), cellular component (CC), and biological process (BP). KEGG is the main database for research on the pathway of related genes and is used to study complex biological functions of genes at the integral level. The transcriptional sequencing results can be downloaded in the GEO database of NCBI with login number: GSE252560.

### RT-qPCR verification

RT-qPCR assay was utilized to validate the reliability of our RNA-Seq data. A total of nine DEGs involved in the auxin, GAs, and ABA pathways were randomly selected. Specific primers were designed using Primer Premier 6 (Supplementary Material Table S1). RNA was extracted by RNA kits (Takara, Beijing, China) from stem segments under different stages, and the cDNA was synthesized using the PrimeScript RT Master Mix kit (Takara). RT-qPCR was performed using TB Green Premix Ex Taq II Kit (Takara). Each reaction was set to three biological replicates. The relative gene expression was standardized using the reference gene *UBCE* (Annotation ID: 109,832,941) and calculated using the 2^−ΔΔCT^ method. The Pearson correlation coefficient (R) between gene expression rates obtained from RNA-Seq and RT-qPCR was calculated using Origin 2022 software.

### Data statistics and analysis

The experimental data were statistically analyzed using Microsoft Excel 365 software. Significance analysis was done using SPSS 26.0 software, and drawing was done using Origin 2022 software.

## Results

### Growth curve and tissue slice analysis of asparagus

**Fig. 1:**
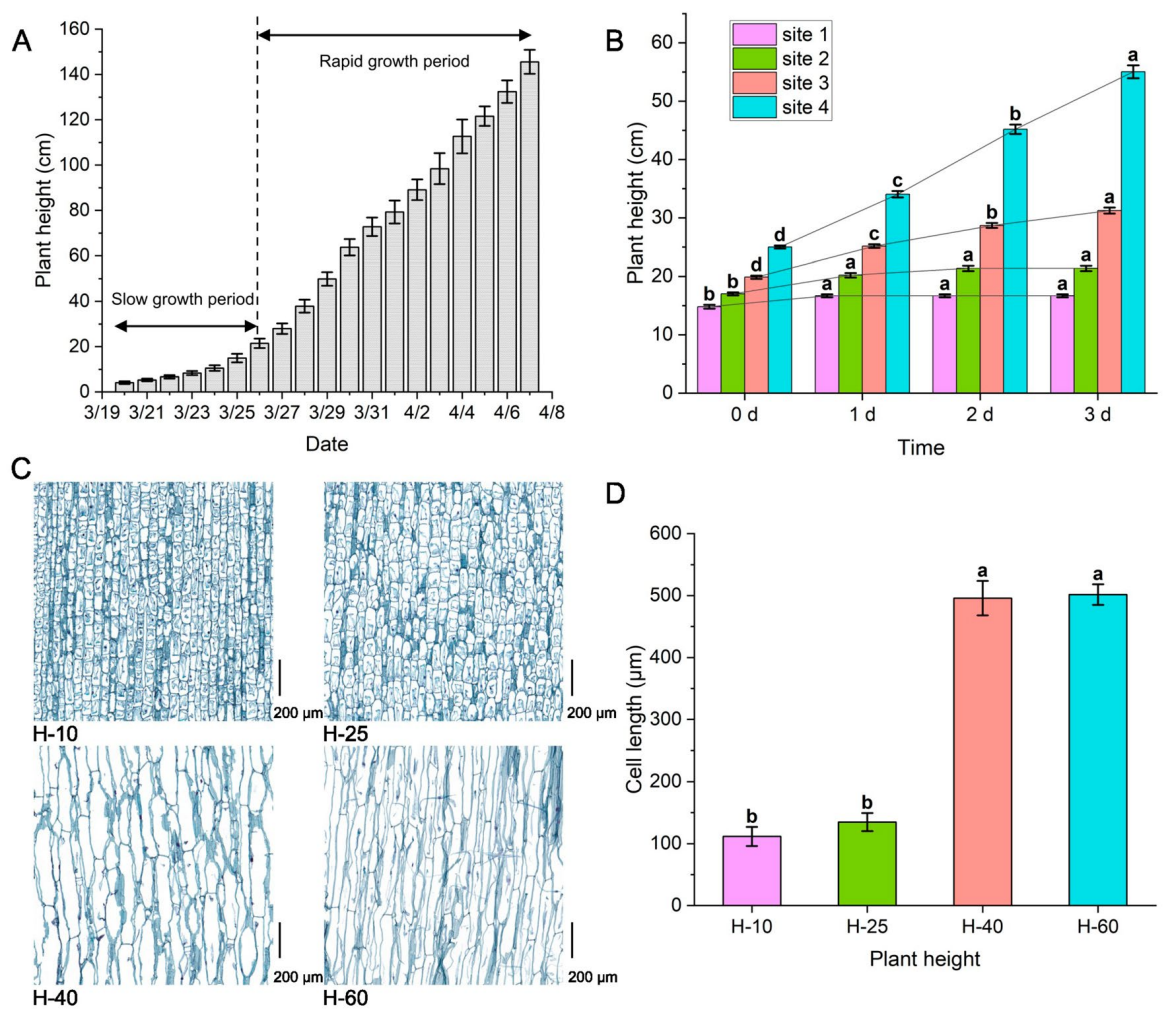
Changes in morphology and cellular structure of asparagus. **A**. Asparagus growth curve. **B**. Displacement map of time and plant height markers. **C**. Asparagus stem longitudinal slice (bottom right corner ruler 200 μm). **D**. Relationship between plant height and cell length. The error bar represents a standard error (SE). Mean values ± SE. Different lowercase letters indicate significant differences among each treatment, *P* < 0.05, the same below

Under suitable growth conditions, asparagus plants exhibit a “slow-fast” exponential growth pattern up to a height of 150 cm (Fig. 1A, Fig. S1), with relatively slow growth from 0 to 20 cm and rapid growth from 20 to 150 cm. We monitored the dynamic changes in plant height of asparagus over three days. Notably, through continuous observation of the 25 cm asparagus marker, it was found that the stem segment from 17.03 to 19.88 cm can increase by 3.46-fold (Fig. 1B). An analysis of the tissue section of asparagus revealed a statistically significant difference in cell length between samples H-25 and H-40. The longitudinal length of cells increased 3.72-fold, measuring 501.73 μm from 134.92 μm (Fig. 1C-D).

### Auxin, GAs, and ABA analysis of asparagus elongation growth

**Fig. 2:**
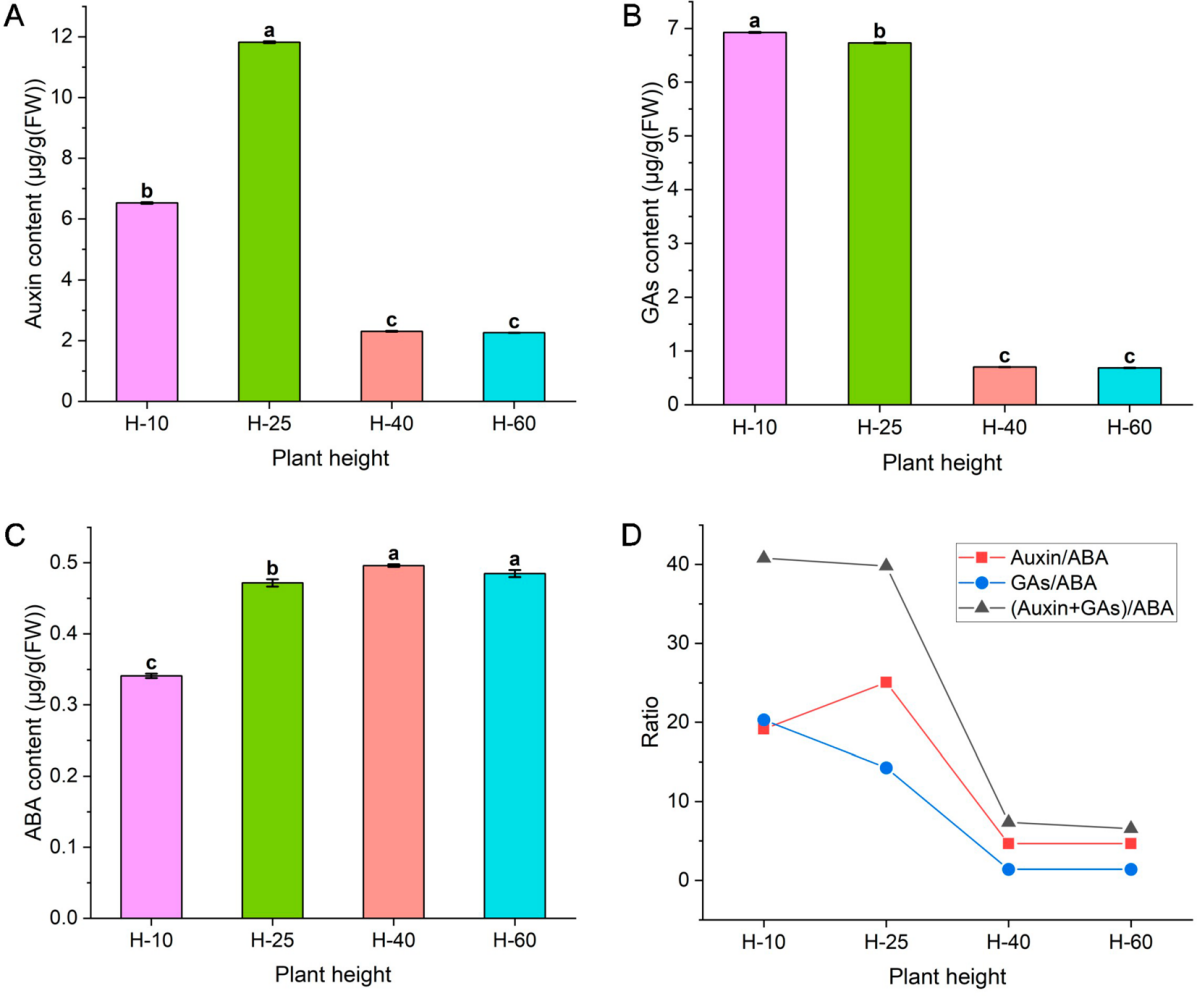
The different Auxin (**A**), GAs (**B**), ABA (**C**) content, and endogenous hormone ratio (**D**) in different stages of growth and development in asparagus stems

As presented in Fig. 2, the auxin content exhibited a unimodal curve change across four stages of growth and development, with a maximum value of 11.82 µg/g (FW) at H-25, significantly higher than the values observed at other growth and development stages (Fig. 2. A). The content of GAs decreases steadily during the four growth and development stages, reaching 6.73 µg/g (FW) at H-25, significantly different from the values observed at other stages of growth and development. The value of H-25 was 9.60-fold that of H-40 (Fig. 2B). The ABA content showed a steady increase across four stages of growth and development, reaching 0.47 µg/g (FW) at H-25, significantly different from other stages of growth and development, which was 0.95-fold at H-40 (Fig. 2C). The relative content of plant hormones is equally important for plant growth and development. The auxin/ABA ratio showed a unimodal curve change across the four stages of growth and development, with a maximum value of 25.54 at H-25, which was 4.29-fold greater than its value at H-40. Ratios of GAs/ABA and (GAs + Auxin)/ABA showed a steady decrease during the four stages of growth and development (Fig. 2D).

### Sugar analysis of asparagus elongation and growth

**Fig. 3:**
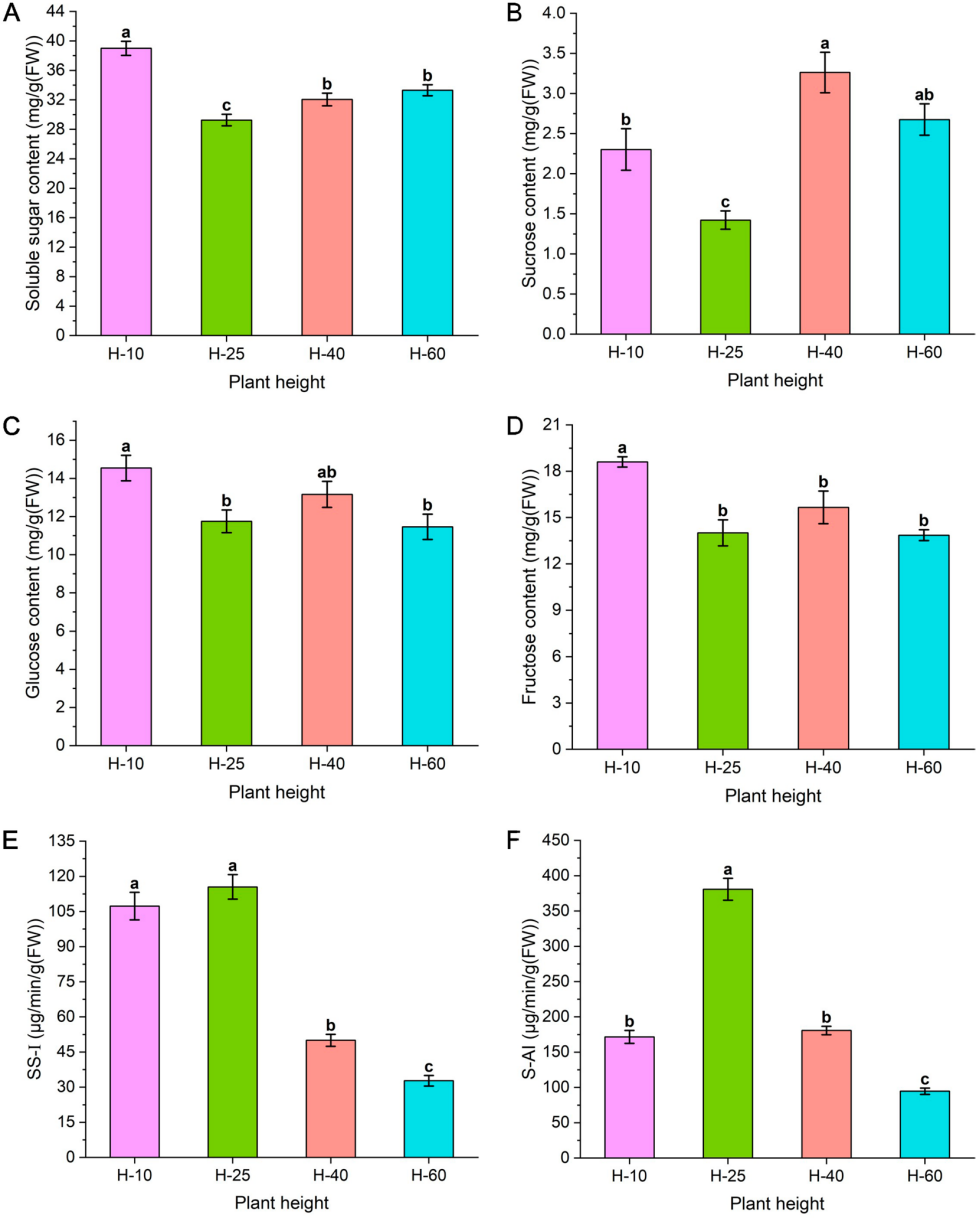
The different soluble sugar (**A**), sucrose (**B**), glucose (**C**), and fructose (**D**) content and SS-I (**E**) and S-AI (**F**) activity in different growth and development stages of asparagus stems

As illustrated in Fig. 3, the total soluble sugar content showed a single valley curve change across all four stages of growth and development, with a minimum value of 29.26 mg/g (FW) at H-25, significantly lower than the values recorded at other stages of growth and development (Fig. 3A). The sucrose, glucose, and fructose content showed a wave shape with an upward opening at the beginning of the four stages of growth and development. The lowest sucrose content was 1.42 mg/g (FW) at H-25, which was 0.44-fold lower than the sucrose content at H-40 and significantly lower than other growth and development stages (Fig. 3B). The glucose and fructose content at H-25 were only significantly lower than those of H-10 and did not significantly differ from those observed during other growth and development stages (Fig. 3C-D). The activity of SS-I (Fig. 3E) and S-AI (Fig. 3F) showed a single peak curve change during all four growth and development stages, with the highest values at H-25. The activity of SS-I was 115.48 µg/min/g (FW) at H-25, significantly higher than that of H-40 and H-60, and was 2.31-fold that of H-40. S-AI activity was 380.88 µg/min/g (FW) at H-25, 2.11-fold than that of H-40, significantly higher than other growth and development stages.

### Transcriptome sequencing and gene expression analyses

**Fig. 4:**
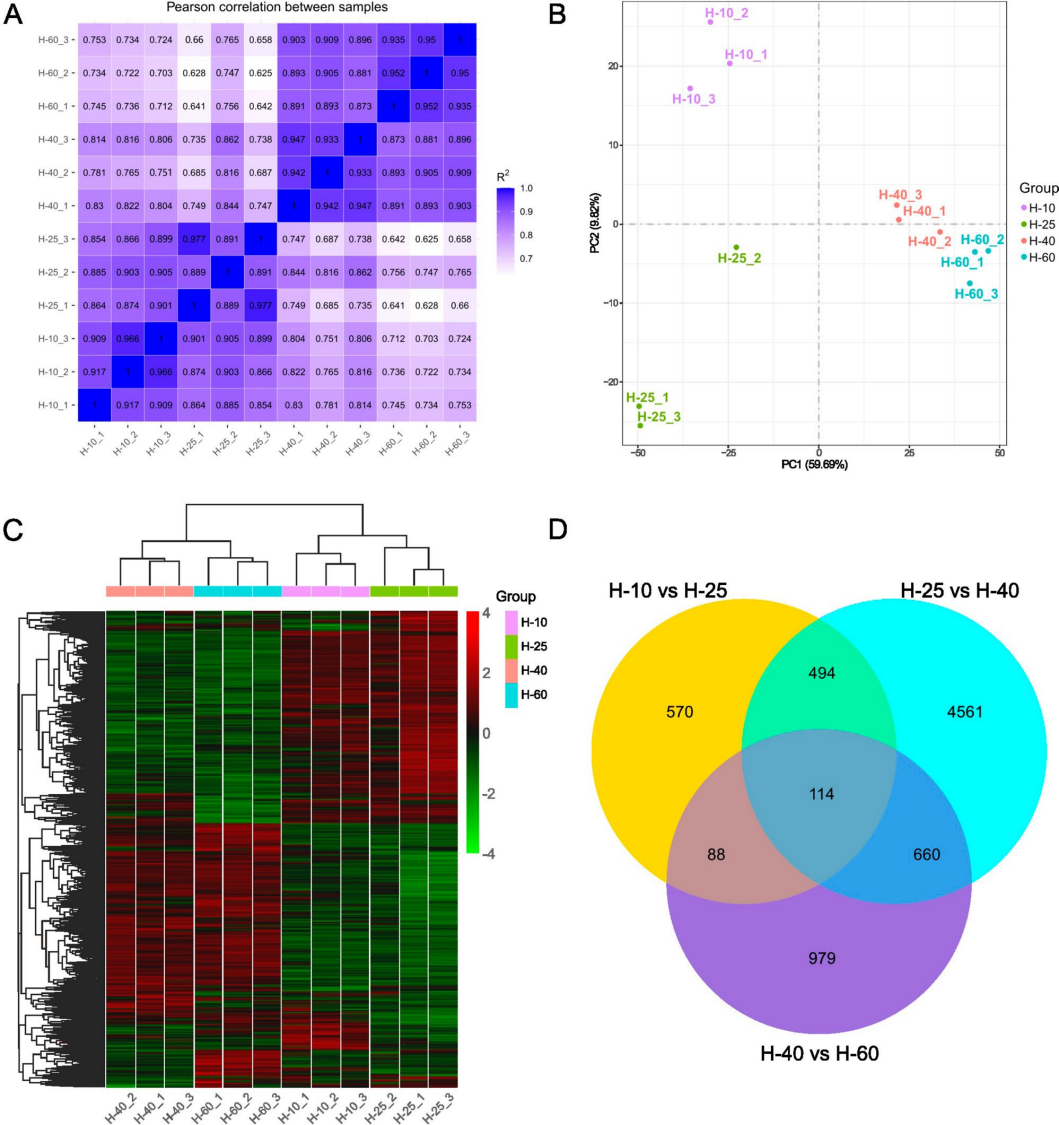
Overview of basic transcriptome information. **A**. Correlation heatmap between samples. **B**. Principal component analysis diagram. **C**. Cluster analysis of differentially expressed genes (the vertical axis stands for relative expression, and the red and green stand for upregulation and downregulation of DEGs, respectively). **D**. Venn diagram of DEGs (comparison of DEGs in H-10 vs. H-25, H-25 vs. H-40, and H-40 vs. H-60)

RNA-Seq technology was used to study the changes in gene expression during the elongation and growth of asparagus stems. Based on the FPKM values of all genes in each sample, the correlation coefficients of intra-group and inter-group samples were calculated and plotted on a heatmap. The intra-group R² ≥ 0.889 (Fig. 4A). PCA analysis showed that samples within the group were densely clustered. In contrast, samples between groups were more dispersed (Fig. 4B). A cluster analysis was performed on the DEGs in four growth and development stages of asparagus and found that H-10 and H-25 were clustered together, whereas H-40 and H-60 were clustered together, which corresponded to the processes of growth and maturity (Fig. 4C). Three comparison groups were established to explore the differences in gene expression under different treatment conditions. The comparison groups were H-10 vs. H-25, H-25 vs. H-40, and H-40 vs. H-60. The number of DEGs was significantly higher in the H-40 group compared to the H-10 group when H-25 was used as the DEG control group. The Venn diagram of DEGs (Fig. 4D) demonstrates 4561 DEGs for H-25 vs. H-40 differently from H-10 vs. H-25 and H-40 vs. H-60.

### Functional annotation analysis of DEGs

**Fig. 5:**
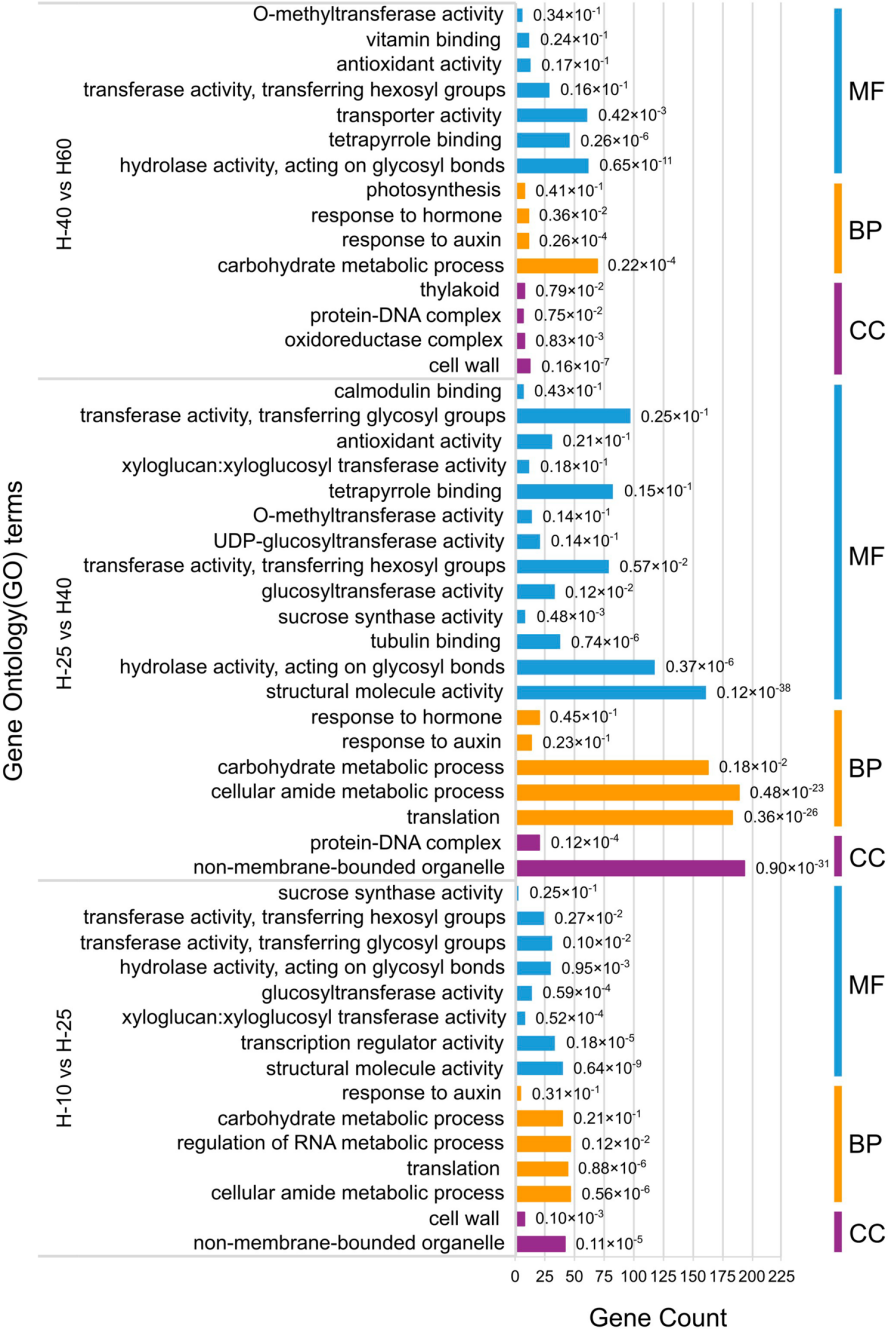
Diagram of GO function annotation and classification in the three comparison groups. The X-axis is gene count, the Y-axis is GO terms, and the padj is on the bar chart

GO enrichment classification was performed for the DEGs with padj ≤ 0.05 (Fig. 5). Most DEGs in H-10 vs. H-25 and H-25 vs. H-40 were enriched in BP and MF, with active cell metabolism in the early stages of asparagus growth and development. A greater number of DEGs were enriched in CC in H-40 vs. H-60, and there were significant changes in cellular substances during the later stages of asparagus growth and development. Most DEGs in the three comparison groups were significantly enriched in BP, including “reactions to auxin”, “carbohydrate metabolism processes”, and so on. They were enriched significantly in MF, mostly related to the hydrolysis and transfer of sugar groups. Certain DEGs for CC were mainly concentrated in the “cell wall”. The DEGs of H-40 vs. H-60 were also enriched in “oxidoreductase complexes,” which could be associated with aging and lignification.

### Transcriptome pathway annotation analysis

**Fig. 6:**
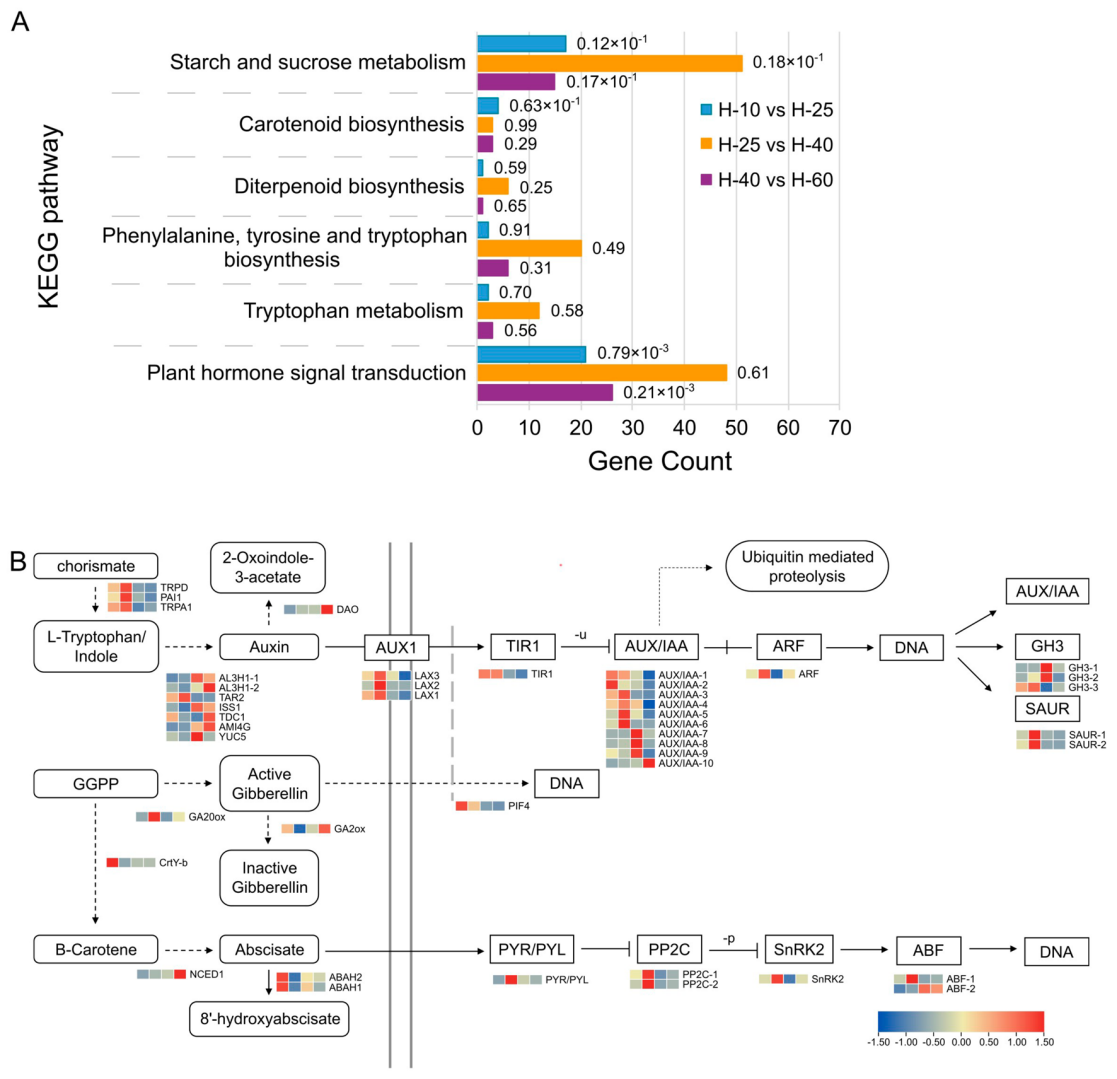
DEGs in KEGG pathway. **A**. Diagram of KEGG pathway in the three comparison groups. Annotation is the same as the GO function annotation diagram. **B**. Schematic diagram of synthesis, metabolism, and action of auxin, GAs, and ABA. Four squares represent H-10/H-25/H-40/H-60 from left to right. The horizontal axis stands for relative expression, and the red and blue stand for upregulation and downregulation of DEGs, respectively

The upregulated and downregulated DEGs were enriched in the KEGG database for metabolic function analysis (enrichment parameter corrected padj ≤ 0.05). KEGG pathways related to “hormone” and “starch and sucrose metabolism” were plotted (Fig. 6A). “Tryptophan metabolism” and “phenylalanine, tyrosine, and tryptophan biosynthesis” are related to auxin. “Diterpenoid biosynthesis” and “Carotenoid biosynthesis” are associated with GAs and ABA, respectively. The number of DEGs in H-25 vs. H-40 was more than in H-10 vs H-25 and H-40 vs H-60.

To investigate the genes involved in the elongation of asparagus tender stems caused by auxin, GAs, and ABA, 44 DEGs were selected from the transcriptome, including protein-coding and transcription factor genes (Fig. 6B). The transcription factor genes included *PIF4* and *ABF*. *TRPD*, *TRPA1*, *TAR2*, and *PAI1* were highly expressed in H-10 and H-25 in auxin synthesis; the auxin degradation gene *DAO* was highly expressed in H-60. The increased expression levels of *LAX1*, *LAX2*, and *LAX3* in H-25 facilitated auxin transport, while downstream genes *SAUR* and *ARF* were also highly expressed. GAs and ABA can be synthesized by GGPP. *GA20ox* was highly expressed at H-25, thereby promoting the synthesis of GAs, whereas *GA2ox* was poorly expressed. An increase in the expression level of *NCED1* at H-60 was observed, which is beneficial for ABA increase. *ABAH1* and *ABAH2* had higher expression levels at H-10, and their encoded products decompose ABA. The expression level of *PIF4* gradually decreases with stem growth and development in GA signal transduction. *PYR/PYL*, *PP2C*, *SnRK2*, and *ABF* were also highly expressed in ABA signal transduction at H-25.

### RT-qPCR analysis

**Fig. 7:**
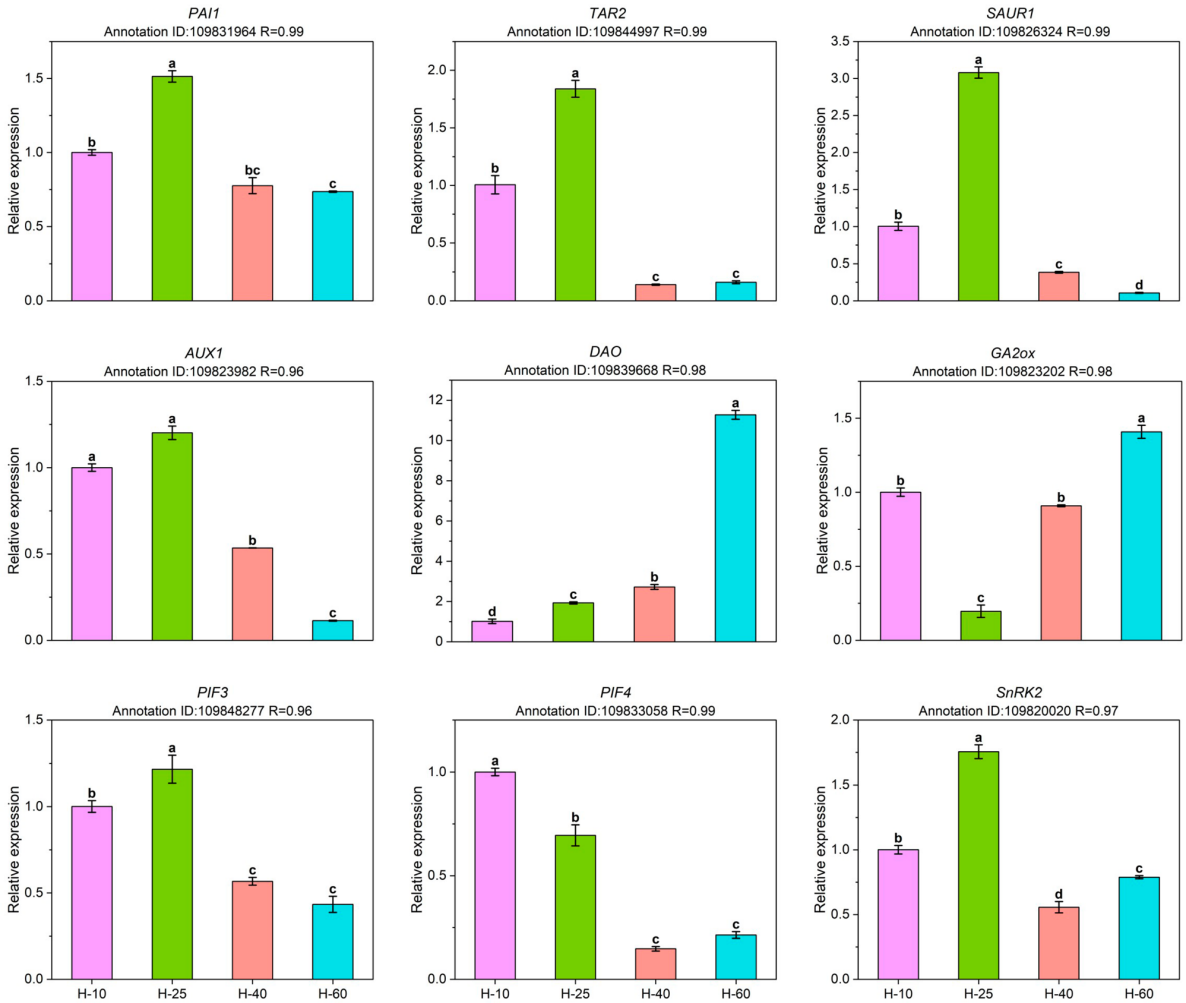
RT-qPCR analysis. The Annotation ID of each DEG is placed at the top of the panel

To verify the reliability of gene expression data obtained from transcriptome sequencing, we conducted RT-qPCR validation on nine genes related to auxin, GAs, and ABA pathways (Fig. 7). The expression trend of DEGs detected by RT-qPCR was consistent with the transcriptome sequencing results. *PAI1* and *TAR2* related to auxin synthesis had high expression levels in the early stage. On the contrary, *DAO*, responsible for degrading auxin, had a high expression level in the later stage. The *AUX1* gene encodes an auxin transporter protein in the cell membrane that facilitates the transport of auxin, which is highly expressed at H-10 and H-25. The extensive *AUX1* expression at H-10 and H-25 is beneficial for the transmission. Low expression of *GA2ox* can reduce the loss of active GAs at H-25. Early expression of *PIF*, a positive regulatory transcription factor of gibberellin, is beneficial for cell length elongation. The *SnRK2* gene is an important component of the ABA signal transduction pathway. The Pearson correlation coefficient (R) between RNA-Seq and RT-qPCR was greater than 0.95, indicating that the latter is more reliable.

### Correlation analysis

**Fig. 8:**
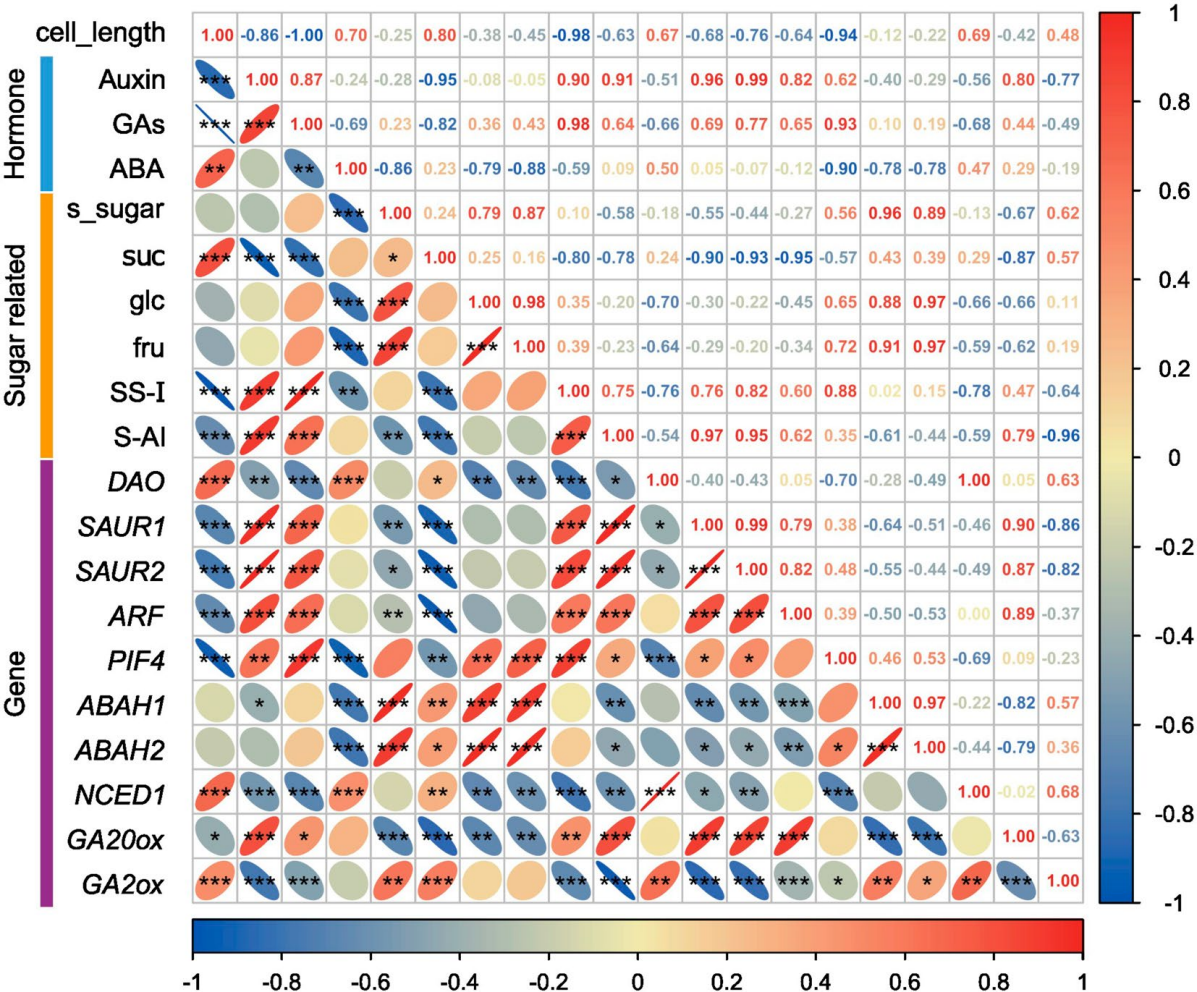
Correlation analysis between multiple parameters. *P < 0.05, **P < 0.01, ***P < 0.001; s_sugar: soluble sugar; suc: sucrose; glc: glucose; fru: fructose

We normalized cell length, hormones (auxin, GAs, and ABA), carbohydrates, SS-I, S-AI, and some genes for correlation analysis (Fig. 8). The research found that among the three hormones, auxin, and GAs are significantly negatively correlated with ABA but auxin and GAs are significantly positive with each other. Auxin and GAs showed a statistically significant negative correlation with cell length, while ABA demonstrated a statistically significant positive correlation with cell length in terms of hormones and cell length. Auxin had a statistically significant positive correlation with *SAUR1*, *SAUR2*, and *ARF*, whereas it had a statistically significant negative correlation with *DAO* regarding hormones and genes. GAs showed a significant positive correlation with *GA20ox* and *PIF4* while a significant negative correlation with *GA2ox*. ABA was significantly positively correlated with *NCED1*, while it had a significant negative correlation with *ABAH1* and *ABAH2*. Regarding hormones and carbohydrates, auxin and GAs had a significant negative correlation with sucrose, whereas they had a positive correlation with soluble sugars, fructose, and glucose. ABA exhibited a positive correlation with sucrose but had a significant negative correlation with soluble sugar, fructose, and glucose. In terms of hormones, SS-I and S-AI, auxin, and GAs showed a significant positive correlation with SS-I and S-AI, while ABA was significantly negatively correlated with SS-I and positively correlated with S-AI.

## Discussion

**Fig. 9:**
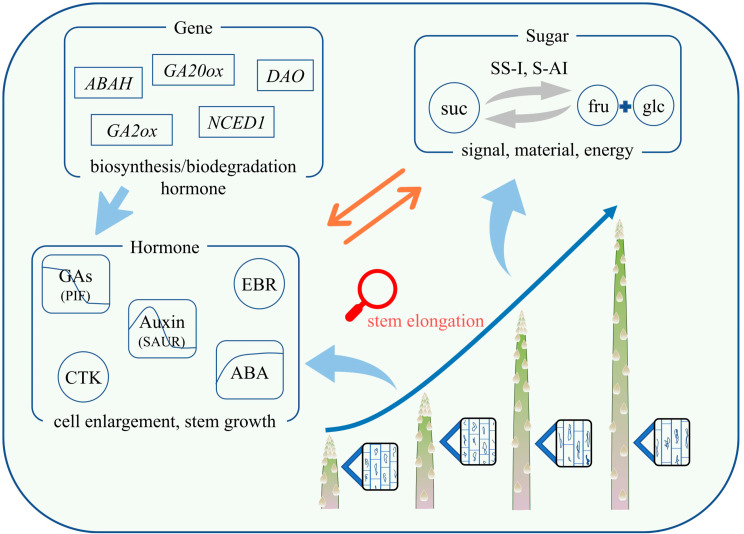
Pattern diagram of the formation process of asparagus tender stems

This study found that the growth curve of asparagus shows a “slow-fast” within 150 cm. When the asparagus reaches a height of 25 cm, the lower 2.85 cm stem segment (17.03–19.88 cm) can extend to 9.88 cm, approximately 3.46-fold. We further investigated and summarized the pathways involving cell morphological structure, sugar, and plant hormones in the elongation and growth of asparagus tender stems. The morphological structure of cells mainly focuses on changes in the longitudinal length of cells. Carbohydrates are more inclined to observe glucose, fructose, sucrose, and their related metabolic enzymes. Plant hormones focus on the three widely studied hormones: auxin, GAs, and ABA (Fig. 9). Furthermore, we attempted to elucidate the internal connections between some factors through correlation analysis.

### Cell morphological structure and sugar regulate the elongation and growth of asparagus tender stem

The elongation and growth process of asparagus tender stems is accompanied by the elongation of active cells, which is similar to the bolting process of Chinese cabbage [23]. Previous studies have found that a decrease in the number and length of cells in the stem is an important cause of dwarfing individuals in barley [24] and elephant grass [25]. At H-25, the 17.03–19.88 cm stem segment was in an active state of elongation and growth. In the sample, the cell length of H-60 is 3.72-fold that of H-25. Stem elongation has been demonstrated to be an external manifestation of cell length extension.

Sugar can serve as both an energy source and a signaling substance in plant growth and development [13]. Both Wei et al. [26] and Wang et al. [27] found that sucrose can increase the elongation rate of bamboo internodes. S-AI may be responsible for decomposing sucrose into glucose and fructose, which raises vacuolar osmotic pressure and induces vacuolar growth and cell division. Highly active S-AI is often associated with the rapid growth of plant young tissues and the rapid expansion of storage organs [28]. Consistent with previous research, this study found that the enzyme activities of S-AI were higher at H-25, indicating that sucrose may be extensively decomposed into hexose for plant utilization. In addition, The reversible decomposition of sucrose by SS-Ι also exhibits high activity in H-25. In the later stage, enzyme activity and sucrose decreased, possibly due to transport to other tissues.

### Auxin, GAs, and ABA regulate the elongation and growth of asparagus tender stems

Auxin controls various aspects of plant growth and development by regulating the basic cellular processes of expansion, division, and differentiation [29]. The synthesis of IAA in Arabidopsis is hindered, resulting in a loss of apical dominance and a decrease in plant height [30]. Hayashi et al. [31] showed that auxin is primarily stored, activated, and inactivated through the GH3-ILR1-DAO enzymatic pathway to maintain homeostasis. This study found that at H-25, the expression of tryptophan/indole synthesis-related genes, *TRPD* and *TRPA1*, was upregulated, while the expression of *DAO* was upregulated at H-60. The changes in auxin content are consistent with the pattern observed in the expression of related genes. Auxin can promote the transcription of auxin-responsive genes, such as *AUX/IAA*, *GH3*, and *SAUR*, with *SAUR* as the main effector factor of auxin that regulates cell growth [32]. By upregulating the expression level of the *SAUR* gene and inhibiting the activity of PP2C. D phosphatase, cell growth is induced through extracellular acidification, cell wall relaxation, and increased phosphorylated H^+^-ATPases [33, 34]. For the *GH3* gene, Zhao et al. [35] found that *MsGH3.5* overexpression in apple variety ‘Malus sieversii Roem’ leads to the development of dwarf phenotypes. This study found that at H-25, genes such as *LAX1*, *LAX2*, *LAX3*, *ARF*, *SAUR-1*, and *SAUR-2* were highly expressed, which can induce the auxin signal. Consistent with previous research, we found that high expression of the *GH3-1* and *GH3-2* genes at H-40 may promote auxin inactivation, cell growth arrest in the stem.

The active GA content in plants is mainly regulated by *GA20ox* [36] and *GA2ox* [37]. The changes in GAs content are consistent with the pattern observed in the expression of related genes. Xiao et al. [38] found that inhibiting tomato *GA20ox1* or *GA20ox2* resulted in shorter stems, reduced internode elongation, and smaller dark green leaves in transgenic plants. *GA2ox* gene overexpression leads to a dwarfing phenotype in switchgrass (*Panicum virtum* L.) [39]. This study found that *GA20ox* gene expression was upregulated at H-25, while that of the *GA2ox* gene was upregulated at H-60. Genetic studies have shown that the six *PIF* family members: *PIF1*, *PIF3*, *PIF4*, *PIF5*, *PIF7*, and *PIF8* promote hypocotyl elongation and growth [40]. Similarly, this study found that the *PIF4* gene was highly expressed at H-25, promoting stem elongation and growth.

ABA is primarily synthesized through the carotenoid pathway. Typically, 9-cis-epoxycarotenoid dioxygenase (NCED) is a key rate-limiting enzyme in synthesizing abscisic acid in plants [41]. According to Zhou et al. [42], *IbNCED1* overexpression in sweet potatoes promotes ABA accumulation and inhibits active GA_3_ content and plant height. This study found that *ABAH1* and *ABAH2* genes were highly expressed at H-10, whereas *NCED* genes were poorly expressed, coordinating ABA content. Particularly, genes involved in the ABA signal transduction pathway are generally highly expressed at H-25. It is speculated that during the peak metabolism period of asparagus, ABA may activate sugar metabolism. The correlation analysis revealed that the relationship between hormones is not an isolated event, but rather an extensive network, consistent with the findings of Gu et al. [43] and Sheng et al. [44]. During stem growth and development, the content of auxin is positively correlated with GAs content, while the content of ABA is negatively correlated with the first two.

### Auxin, GAs, and ABA regulate sucrose metabolism

There are transduction pathways in plants that correlate sugar, hormones, and other nutritional signals [45]. McAdam et al. [46] found that the auxin synthesis of the pea *tar2-1* mutant was impaired and inhibited embryonic growth and sucrose distribution, producing shrunken seeds. The research results of Robert and Friml [47] indicated that the main role of auxin in sucrose metabolism is to promote sucrose decompose. Its mechanism of action may be through the activity of ATPase on the cell membrane to regulate cell turgor and long-distance transportation of sucrose, achieving accumulated photosynthetic products in the storage organs and exogenous IAA application sites. Previous studies have demonstrated that GA_3_ can regulate the synthesis of key enzymes and protein carriers that transport sugars during sugar metabolism [48]. Xu et al. [49] reported that GA could reduce the activity of carbohydrate metabolic enzymes, including SS and ADP-glucose pyrophosphorylase (AGPase), and the expression level of its coding genes, achieving an inhibitory effect on sucrose-induced physiological activity [50]. Cole et al. [51] treated the elongating internodes of alfalfa with GA, sucrose rapidly degraded into hexose, constructing a concentration gradient favorable for sucrose input into the reservoir cells. The findings of Du et al. [52] indicated that GA treatment of early grape fruits results in more active sucrose metabolism and utilization in berries. ABA has a synergistic effect with sucrose and enhances the expression of sucrose-induced genes [50]. Additionally, ABA can regulate the expression of sugar-responsive genes through the downstream signaling element ABI4, thereby regulating sugar metabolism processes [53]. This study found that auxin and GAs had a statistically significant negative correlation with sucrose content and a statistically significant positive correlation with SS-I and S-AI. Auxin and GAs may promote sucrose decomposition and fulfil the growth needs of asparagus tender stems, causing elongation. ABA is significantly negatively correlated with SS-I.

## Conclusions

This study found that auxin and GAs positively regulate the elongation and growth of asparagus stems through synthesis and metabolism, whereas ABA has the opposite effect. Sucrose is decomposed and utilized through enzyme interactions such as SS-I and S-AI, providing energy and serving as signaling substances. Further research is demanded on the mechanism of action and mutual regulatory network of different hormones and sugars in the elongation and growth region of asparagus stems.

### Electronic supplementary material

Below is the link to the electronic supplementary material.

Supplementary Material 1

Supplementary Material 2

## Data Availability

The data discussed in this publication have been deposited in NCBI’s Gene Expression Omnibus (Edgar et al., 2002) and are accessible through GEO Series accession number GSE252560(https://www.ncbi.nlm.nih.gov/geo/query/acc.cgi?acc=GSE252560). To review GEO accession GSE252560: Go to https://www.ncbi.nlm.nih.gov/geo/query/acc.cgi?acc=GSE252560. Enter token qzypwiiojnsrzmh into the box.
