# Supplementary figures and images for: Physiological and transcriptome analysis of changes in endogenous hormone and sugar content during the formation of tender asparagus stems
Source: BMC Plant Biol. 2024 Jun 19;24:581. doi: 10.1186/s12870-024-05277-0 (PMC11186092; doi:10.1186/s12870-024-05277-0)

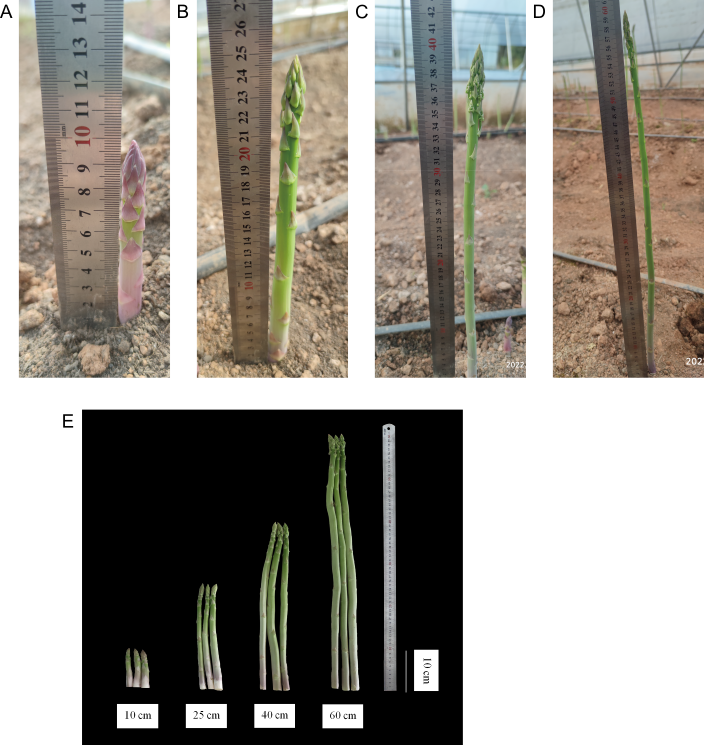


Fig. S1. Asparagus plant height A: 10 cm; B: 25 cm; C: 40 cm; D: 60 cm; E: Schematic diagram of different plant heights

Supplement: Supplementary file 2 — Supplementary Material 2 [file 12870_2024_5277_MOESM2_ESM.docx]
